# Dietary patterns and the risk of rhinitis in primary school children: a prospective cohort study

**DOI:** 10.1038/srep44610

**Published:** 2017-03-15

**Authors:** Xudong Liu, Claudie Chiu-Yi Wong, Ignatius T. S. Yu, Zilong Zhang, Lixing Tan, Arthur P. S. Lau, Albert Lee, Eng Kiong Yeoh, Xiang Qian Lao

**Affiliations:** 1 JC School of Public Health and Primary Care, The Chinese University of Hong Kong, Hong Kong SAR, China; 2 Hong Kong Occupational and Environmental Health Academy, Hong Kong SAR, China; 3 Division of Environment, Hong Kong University of Science and Technology, Hong Kong SAR, China

## Abstract

This study was to investigate the association between dietary patterns and rhinitis in primary school children. 1,599 students without rhinitis at baseline survey were selected from a primary school children cohort. Information on food consumption, respiratory symptoms, and confounders was collected using questionnaires. Dietary patterns were defined using principal component analysis. Logistic regression was performed to calculate odds ratio (OR) with 95% confidence intervals (95% CI). The incidence of rhinitis during 12 months follow-up was 21.2%. Three patterns were extracted and labeled as pattern I, II and III. Dietary pattern II which had higher factor loadings of legumes, butter, nuts and potatoes was associated with an increased risk of rhinitis (OR: 1.34, 95% CI: 1.01–1.87) when the highest tertile of pattern score was compared to the lowest tertile, after adjusted for confounders. Besides, every 1-unit increase of score of pattern II was also associated with an increased risk of rhinitis (OR: 1.19, 95% CI: 1.05–1.35). Neither pattern I nor Pattern III was observed to be associated with risk of rhinitis. A diet with higher levels of consumption of legumes, butter, nuts and potatoes may increase the risk of allergic rhinitis in primary school children.

Asthma and atopic respiratory disorder have become more prevalent worldwide, affecting nearly 235 million people^1^,^2^. It is estimated that 14% of the world’s children were likely to have had asthmatic symptoms^2^. The high prevalence of asthma has been associated with an increase in atopic sensitization, and is paralleled by similar increases in other allergic disorders such as rhinitis and eczema. Asthma has created a heavy global burden and affected the quality of human life significantly, especially in children^3^,^4^. Allergic rhinitis is defined as inflammation of the nasal mucous membrane that appears after the exposure to a particular allergen other than an infection. Although it has received less attention than asthma in epidemiological studies, allergic rhinitis is recognized as the most common allergic manifestation in children and it is highly correlated with asthma^5^,^6^.

Various factors have been reported to be related to asthma and allergy disease in children. In particular, increasing evidence has highlighted a crucial role for both maternal and childhood food exposure as protection against the development of allergic diseases^7^. A considerable number of epidemiological studies have reported the protective effects by intake of individual nutrients^8^,^9^ and individual food item^9^,^10^,^11^,^12^ during later childhood. Dietary pattern which represents a whole profile of a diet might be more useful to study the association between diet and health outcome, because dietary pattern can in some degree reflect the interactions among different foods or different nutrients^13^,^14^. Current study found that both indexed-based patterns, such as the Mediterranean diet pattern^15^,^16^,^17^,^18^, and patterns defined by using posterior approaches^19^,^20^,^21^,^22^ might have impacts on asthma and other allergic sensitization. However, studies on the association between Mediterranean diet and rhinitis in school-aged children did not obtain conclusive results: for example, in adherence to a Mediterranean dietary pattern was inversely associated with rhinitis and other atopic disease in Mexican children^15^ and Greece children^23^, whereas such association was not observed in Turkey children^24^,^25^. Conflicted results were also observed on the association of Mediterranean diet during pregnancy and children rhinitis^26^.

Empirical dietary patterns have mostly commonly defined by using principle component analysis, factor analysis and cluster analysis. Unlike the index-based patterns which paly a particularly important role in evaluating dietary guidelines and recommendations, dietary patterns defined by using posterior data-driven method can reflect the real nutritional or dietary information within a population in a certain period^27^. However, in children, there was only limited evidence on the association between rhinitis and posterior dietary patterns^21^. We, therefore, conducted a prospective cohort study to investigate the association between dietary pattern defined by posterior approach and risk of rhinitis in school-aged children.

## Methods

### Study setting and participants

**Figure 1:**
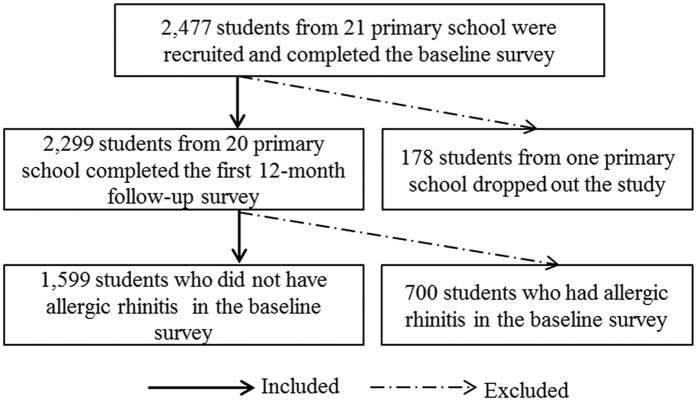
The flow chart of participants’ selection. Solid arrow represents the process of including students; dashed arrow represents the process of excluding students.

The study is based on an ongoing longitudinal prospective cohort in Hong Kong primary school children. The details of this study have been described elsewhere^28^,^29^,^30^. Briefly, twenty-one primary schools were randomly selected with a wide geographical coverage in Hong Kong. To facilitate the spirometry test and the follow-up, students from grades two to four were invited to participate in the study and a total of 2,477 students were recruited in the baseline (Fig. 1). Students were followed up annually for a period of two years after their recruitment. One school (178 students) withdrew during the follow-up. At the end of 2014, 2,299 students from the 20 schools had successfully completed both the baseline and the first-round follow-up surveys, with one year apart. The general characteristics (including age, sex, and education of parents) were similar between the drop out students and the students completed the follow-up survey. Among the 2,299 students, we excluded the 700 (30.4%) students who had rhinitis in the previous 12 months prior to the baseline survey. Thus a total of 1,599 (69.6%) students who did not have allergic rhinitis in the baseline survey and who completed both the baseline and the first follow-up survey were then included in the present analysis. The Joint Chinese University of Hong Kong–New Territories East Cluster Clinical Research Ethics Committee approved this study (Approval ID: 2013.193). This study met the requirements of the Declaration of Helsinki and a written informed consent for each student was signed by their parents.

### Data collection

A structured questionnaire was used to collect a wide range of information during the previous 12 months, including social-demographics, respiratory symptoms, self-reported doctor-diagnosed respiratory diseases, and factors affecting air quality in the home environment. The items in the questionnaire were mainly adopted from the survey tools of the International Study of Asthma and Allergies in Childhood (ISAAC), American Thoracic Society, and the European Community Respiratory Health Survey^31^,^32^,^33^. A simple food frequency questionnaire used in an ISAAC phase three study^34^ was also adopted to collect information on the habits of dietary consumption during the past 12 months. The food items in the question were suitable for children in different regions, including children in Hong Kong^35^,^36^. Parents were required to answer the questionnaire together with their children at home. We also performed a pilot study on 77 children with two-week interval to test the reproducibility of food frequency questionnaire. A moderate association (spearman correlation coefficients ≥0.77) and a moderate inter-rate agreement (kappa statistics ≥0.40) were observed according to suggestion by DeVellis^37^ and Altman^38^. In addition to filling in the questionnaires, each student received a simple health examination to measure his/her weight and height at school in baseline and follow-up surveys.

### Rhinitis ascertainment

Rhinitis, referring in particular to non-infectious rhinitis in the present study, was defined as affecting those children who have “ever had nasal symptoms such as nasal blockage, sneezing, and a runny nose as well as itching eyes or lachrymation in the absence of a common cold in the previous 12 months”. Those who responded “Yes” were further asked one additional question to confirm their response: please indicate the months when your child suffered from rhinitis with 12 month options provided. We compared the responses to two questions. Only those children whose responses to two questions were consistent were deemed to have rhinitis during the 12-month follow-up.

### Dietary exposures

The question “Generally, how often have you consumed the following food groups in the past 12 months?” was used to collect information on dietary intake. A total of 13 groups of food were used in the simple food frequency questionnaire: meat (including pork, beef, lamb, chicken, duck and *et al*.), seafood (including fish, shrimp and *et al*.), fruit (including citrus and non-citrus fruits), vegetables (including leaf vegetables, tuber vegetables, gourd and solanaceae vegetables, green vegetables and *et al*.), legumes (including soy, beans, peas and their products), cereals (including cereals products), rice (including rice products), butter (including margarine), nuts (including peanuts, true nuts and *et al*.), potatoes, milk, eggs and fast food (such as McDonalds, KFC and Subway). The questionnaire assessed weekly consumption using a three-level scale: less than once per week (<1/week), once to twice per week (<3/week) and thrice or more per week (≥3/week).

### Potential confounding factors

Potential confounders collected from the questionnaire included social-demographic characteristics (age, gender, education level of parents, and average size of house for each member), factors affecting air quality in the home environment in the past 12 months (whether there were plants, pets, incense or mosquito coils burning, new furniture or home renovation, carpet, and mold observation), passive smoking, family history of atopic disease (asthma, allergic rhinitis and eczema), other respiratory diseases (defined as self-reported doctor-diagnosed eczema, bronchitis, bronchiolitis, pneumonia, asthma and wheezing). Weight and height were used to calculate body mass index (kg/m^2^). Additionally, the concentration of particles matters less than 2.5 micrometers in diameter (PM_2.5,_ μg/m^2^) on the school campus was measured in the baseline survey using a Dust Trak (TSI) aerosol monitor. To address seasonal variation, we conducted two measurements; one was in the cool season (winter or spring) and the other in the warm season (summer or autumn). The average concentration of PM_2.5_ in each school was used in the present analysis. The total chemical burden score was defined to indicate cumulative weekly exposure level to the 14 types of chemical cleaning products for each participant and was calculated by the method describe in our previous report^39^. The information of weekly usage frequency and average duration of each use were collected by using questionnaire; then total chemical burden score was calculated by using the following formula: 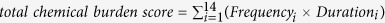, where *Frequency* refers to the weekly frequency of usage of a certain chemical product, *Duration* refers to the average duration of each use, and ***i*** represents the specific chemical cleaning product.

### Statistical analysis

All statistical analyses were performed using R-software (version 3.1.3). *P*-values were derived from two-sided statistic tests and less than 0.05 was considered to be statistically significant.

In order to derive the dietary patterns of the students from the categorical responses of food intake frequency, a simple score for the intake frequency of each kind of food was defined (1 = less than once per week, 2 = once to twice per week, 3 = at least three times per week). The dietary patterns were then extracted using the principal components analysis (PCA) [“psych” package] based on 13 groups of food. Orthogonal (varimax) transformation was adopted to achieve a simple structure with greater interpretability. To determine the number of factors to retain, eigenvalues (>1.0), the scree plot construction, Kaiser–Meyer–Olkin measure of sampling, Bartlett’s test of sphericity, and the interpretability of the factors were considered^40^. The dietary pattern score of each student was calculated using the regression method. Food items with absolute rotated factor loadings ≥0.53 are referred to as “dominant components” hereafter. The labeling of factors was based on our interpretation of the data. A positive loading for a food item indicated a direct association with the pattern, whereas a negative loading suggested that food contributed inversely to the pattern. The scores of dietary patterns were then reverted to categorical variables using the tertile method.

Logistic regression [“aod” package] was performed to assess the relationship between the dietary factors (including individual food items and dietary patterns) and rhinitis. Unadjusted relative risk (OR) and adjusted OR with 95% confidence intervals (95% CI) were calculated in the univariate and multivariable models, respectively. In multivariable model, we firstly adjusted for only the potential confounders, and then adjusted for both confounders and three dietary patterns. In addition, the categories of food or dietary pattern score were included as numerical variable in the logistic model to test for linear trend. In order to ascertain whether there was any interaction between dietary patterns and gender or and age, we included the term “dietary pattern score × gender” and “dietary pattern score × age” in the models separately. However, no interaction was observed (all *P* > 0.05). Students were categorized into subgroups based on gender (male, female) or median of age (≤9 years, >9 years) and stratified analyses were performed. Sensitivity analysis was done by removing those who had other respiratory diseases and by removing those who had family history of atopic diseases.

## Results

During the follow-up period of 12 months, the incidence of rhinitis was 21.2% (339). Besides, 270 students had other respiratory disease (90 had bronchitis, 28 had bronchiolitis, 36 had pneumonia, 125 had eczema and 51 had wheezing). The mean (S.D.) age of the 1599 students was 9.0 (1.0) years, ranging from 7.8 to 14.0 years. No significant difference in age was observed between genders (*p* > 0.05). The general characteristics of the students are presented in Table 1.

In comparison with the rice consumption of less than once per week, the rice consumption of thrice or more per week was associated with a reduced risk of rhinitis (OR: 0.23, 95% CI: 0.09–0.55, *P*_-trend_: 0.016) after adjusting for all potential confounders as well as 13 groups of foods (Table 2). Other 12 foods were not observed to be associated with risk of rhinitis.

Supplementary Table S1 presents the rotated factor loadings using principle component analysis. Three factors were defined. The first factor was characterized with a higher factor loading of meat, seafood, fruit, vegetables, cereals, rice, milk and eggs, explaining about 40% of total variance, labeled as Pattern I. The second factor was characterized with a higher factor loading of legumes, butter, nuts and potatoes, explaining about 16% of the total variance, labeled as Pattern II. The third factor was characterized with a higher factor loading of fast food, explaining about the 9% of the total variance, labeled as Pattern III.

Table 3 presents the relationship between dietary patterns and the risk of rhinitis. After adjusting for potential confounders, every 1-unit increase of score of pattern II was significantly associated with an increased risk of rhinitis (OR: 1.19, 95% CI: 1.05–1.35). By comparing the highest tertile with the lowest tertile of pattern scores, pattern II was also associated with an increased risk of rhinitis (OR: 1.34, 95% CI: 1.01–1.87). Neither pattern I nor Pattern III was observed to be related to the risk of rhinitis. Besides, when considering confounders and all of three patterns, simultaneously, no significant change in effects was observed for all of three patterns.

In sensitivity analysis, similar results were obtained when the analysis was done by removing students who had other respiratory diseases (Supplementary Table S2) and by removing those who had family history of atopic diseases (Supplementary Table S3). In stratified analysis based on the median of the age (Supplementary Table S4) and on the gender (Supplementary Table S5), similar results were observed. Every 1-unit increase of score of pattern II was significantly associated with an increased risk of rhinitis, though the effect in females was marginally significant.

## Discussion

In this prospective cohort study, we studied the association between dietary patterns defined by principle component analysis and risk of allergic rhinitis in school-aged children. Our results show that dietary pattern II which had a higher factor loading of legumes, butter, nuts, and potatoes was associated with an increased risk of rhinitis. No significant association was observed for other two dietary patterns.

This study found that both nuts and potatoes consumption with thrice or more per week was associated an increased risk of rhinitis. This was consistent to the results form ISAAC Phase Three study which reporting that both nuts and potatoes consumption of thrice or more per week were associated with increased risk of both rhinoconjunctivitis and severe rhinoconjunctivitis in children aged 13–14 years^35^. In contrast, rice consumption was related to the decreased risk of rhinitis in our study, this was consistent to the results from a cross-sectional study in Turkey schoolchildren^41^ and from ISAAC Phase One study^36^. We observed that rice intake of ≥3 times/week had OR of 0.63 and rice intake of 1~2 times/week had OR of 0.43. The possible reason for this may be due to the limited sample size in each category. However, when we considering the effects of both confounders and other groups of food, the significant association with was only observed in rice, which playing a protective role. This indicates that there might have some joint effects or complicated cross-reactivity among different groups of food. Using single-food approach is valuable for understanding potential biological mechanisms underlying observed associations, but it is limited by the multicollinearity of dietary intake variables and the inability to detect small effects of single dietary components; however, the cumulative effects of multiple dietary ingredients included in a dietary pattern may be sufficiently large to be detectable.

Our study used principle component factor analysis to define dietary patterns and found that dietary pattern II which had higher factor loadings of legumes, butter, nuts and potatoes was associated with an increased risk of rhinitis in school-age children, no matter pattern score as continuous variable or as categorical variable. Consistent results were observed in sensitivity analysis and stratified analysis, though the effect in females was marginally significant. Possible mechanism maybe due to that food-induced allergic reactions are responsible for a variety of symptoms involving the skin, gastrointestinal tract, and respiratory tract and might be caused by IgE-mediated and non–IgE-mediated (cellular) mechanisms^42^. A minority of food, including peanut, tree nuts, soy, was thought to cause the majority of allergic reactions^43^. Sensitization, mainly to peanuts, is occurring in very young children, and multiple peanut/nut allergies appear progressively^44^. Leguminous crops are also reported to be source of IgE mediated reactions in Mediterranean and Asian countries^45^. People with soybean allergy sensitized to Gly m 5 or Gly m 6 allergens may be at greater risk of experiencing severe allergic reactions^46^. In addition, cross-reactivity was also observed among different legumes, between legumes and nuts and between legumes and other allergen^47^. Butter is extracted from animal fat and it has an abundance of saturated fatty acids. A high intake level of saturated fatty acids may modify serum cholesterol levels and influence the arachidonic acid in the cell membrane^48^. Both serum cholesterol and arachidonic acid can affect lymphocyte function, which further lead to reinforcement in bronchial reactivity and reversible airway obstruction^49^. Margarine was included in butter in this study. Margarine consumption was also reported to be associated with allergic sensitization in children^50^,^51^. Margarine contains lots of linoleic acid which is a precursor of PGE2, which in turn may promote allergic sensitization by inhibiting the formation of IFN-γ^52^,^53^. Potatoes generally contains a higher level of protein than other tubers or cereals, and up to 40% of the protein could be patatin, which belongs to a family of immunological identical isoforms of glycoproteins and are responsible for allergic reactions in children^54^,^55^. We observed that, when taken pattern scores as categorical variables, no significant association was observed in both two groups based on median of age and based on genders. This might due to the smaller sample size in subgroup analysis.

In contrast, dietary pattern I, which had a higher factor loading of meat, seafood, fruits, vegetables, cereals, rice, milk and eggs, was not significantly associated with any risk of rhinitis. Regarding the individual groups of food included in pattern I, there are a number of studies which investigated their relationships with rhinitis, but the majority of these studies had a cross-sectional design and the results were inconsistent^25^,^56^,^57^,^58^,^59^,^60^. Dietary pattern III, which had a higher factor loading of fast food, was not related to any risk of rhinitis. This was consistent to results from above analysis on the single group of food. ISAAC Phase Three study reported that the consumption of fast food with thrice or more per week was associated increased risk of rhinitis and other allergic diseases^35^. The conflicted results might be due to the variance of dietary habits and dietary preference.

In comparison with other study with factor analysis or principal component analysis, two dietary patterns were extracted by using factor analysis in cross-sectional study in Taiwan school-age children^21^. Both patterns were observed to be associated with and increased risk of allergic rhinitis, However, this study^21^ had some conflicts itself from the information presented: high-protein, high-fat, and western diet pattern was associated with increased risk of allergic rhinitis when score as the continuous variables but not when as categorical variables; the health pattern was seemed to be associated with an increased risk of rhinitis when pattern score takes as both continuous and categorical variables whereas the author did not provide any reason.

Food-induced allergic rhinitis is also occurs together with other food allergy symptoms such as asthma, eczema, oral allergic manifestations^61^. Persistent wheezing also was report to be a risk factor in the development of allergic rhinitis in children after five years of age^62^. In this study, consistent adverse effect by pattern II was obtained after removing the children with other respiratory diseases. Family history of atopic disease might influence the association^63^. However, in this study, similar adverse effect by pattern II were obtained after removing those children with family history of atopic disease, which indicate that the pattern effect did not attribute to the effects of family history of atopic disease.

Dietary pattern is a better and more comprehensive approach, by which the holistic effects of diet can be explored. PCA is a realistic approach to identifying distinct dietary patterns which represent the whole profile of the diet consumed^13^. Besides, dietary patterns by the principle component analysis have ability to overcome issues pertaining to the multicollinearity of food, nutrients, and other dietary constituents, and the cumulative effects of multiple dietary ingredients included in a dietary pattern may be sufficiently large to be detectable^64^. By using PCA, three dietary patterns were defined in this study. The overall value of Kaiser–Meyer–Olkin was 0.94 and *p* value for Bartlett’s test of sphericity was less than 0.001, suggesting a proper sample size with which to perform PCA^40^. The individual measures of sampling adequacy for each item ranged from 0.86 (nuts) to 0.96 (seafood), ensuring an acceptable level of accuracy of measurement for both patterns in our study. The communality for each component was large than 0.50, which means that the factor loadings derived from principal component analysis can be used for further analysis.

The strengths of the present study include the prospective cohort study design, relatively large sample size, high response rate and adjustment for a wide range of confounders. Another advantage is that the reproducibility test showed there were moderate association and moderate agreement for the food frequency questionnaire. All students were survey in the same season in both baseline survey and follow-up survey. We also examined the relationship between outcomes and baseline dietary consumption by using same analytic approach: three similar patterns were extracted; pattern II was associated with an increased risk of rhinitis when adjusted for confounders (OR: 1.44, 95% CI: 1.06–1.95, *p*-trend: 0.020) and adjusted for confounders as well as three patterns (OR: 1.45, 95% CI: 1.07–1.97, *p*-trend: 0.021) when comparing the highest tertile with the lowest tertile of pattern scores. The consistent results were sufficiently close to make the validity of our questionnaire within an acceptable range.

However, there are limitations. Firstly, the food frequency questionnaire was applied over last 12 month, which might lead to somewhat recall bias. However, the reproducibility test proved higher consistent in the responses to each group of food. Secondly, multiple tests for the 13 food items might result in false-positive associations, but the results for individual groups of food prompted us to conduct dietary pattern analysis to surmount the shortcomings of traditional approach based on single dietary ingredient. Thirdly, the intake amount of each food was not collected and the total energy intake was not available, which is similar to other previous studies^48^,^57^. This limitation rendered us to take them into account directly. In this study, we made adjustments for body mass index and weekly physical activity which were suggested as an indirect alternative approach^27^,^65^. Fourthly, there were only 13 groups of food were included in the questionnaire. Fewer food groups used in the pattern analysis may reduce the study power. A more detailed and comprehensive food frequency questionnaire should be developed in future studies to warrant more in-depth and thorough analyses. Finally, information on rhinitis was retrieved from questionnaire and it refers to non-infectious rhinitis. We were not able to exclude non-allergic rhinitis such as vasomotor cases in the present study. However, the proportion of non-allergic rhinitis within non-infectious generally is very low in children and should not affect our results.

In conclusion, this study found that a diet pattern with higher levels of consumption of legumes, butter, nuts and potatoes may increase the risk of rhinitis in primary school children. Results from this study suggest that prevention strategies should consider not only single dietary ingredients but also complex dietary patterns.

## Additional Information

**How to cite this article:** Liu, X. *et al*. Dietary patterns and the risk of rhinitis in primary school children: a prospective cohort study. *Sci. Rep.* **7**, 44610; doi: 10.1038/srep44610 (2017).

**Publisher's note:** Springer Nature remains neutral with regard to jurisdictional claims in published maps and institutional affiliations.

## Supplementary Material

Supplementary Information

## Figures and Tables

**Table 1:** Basic characteristics of the study subjects in baseline survey.

Items	Value			
	Total	Non-Rh*	Rh*	P value
	Mean (standardized deviation)			
Age, years	9.0 (1.0)	9.0 (0.9)	9.0 (1.0)	0.557†
Body mass index, kg/m^2^	17.3 (3.1)	17.3 (3.1)	17.3 (3.2)	0.990†
Average size of house for each member, m2	9.4 (4.1)	9.4 (4.1)	9.6 (3.8)	0.363†
Concentration of PM2.5 in each school (μg/m2)	54.9 (42.0)	55.0 (40.6)	54.6 (46.9)	0.557†
	Median (Interquartile)			
Total chemical burden score	3.2 (2.2)	3.1 (2.2)	3.4 (2.6)	0.233‡
	Number (Percentage %)			
Gender, male	738 (46.2)	578 (45.9)	160 (47.2)	0.709§
Passive smoking at home, yes	316 (19.8)	246 (19.5)	70 (20.6)	0.700§
Keeps a pet at home, yes	151 (9.4)	118 (9.3)	33 (9.7)	0.919§
Has a plant at home, yes	709 (44.3)	565 (44.8)	144 (42.5)	0.474§
Burns incense/mosquito coils at home, yes	362 (22.6)	293 (23.3)	39 (20.4)	0.289§
Has new furniture or renovation at home, yes	357 (22.3)	282 (22.4)	75 (22.1)	0.943§
Has carpet at home, yes	257 (16.1)	204 (16.2)	53 (15.63)	0.870§
Has mould at home, yes	574 (35.9)	445 (35.2)	129 (38.1)	0.375§
Weekly physical activity				0.629§
Never/less than once	450 (28.1)	353 (28.0)	97 (26.6)	
Once or twice	866 (54.2)	678 (53.8)	188 (55.5)	
At least three times	283 (17.7)	229 (18.2)	54 (15.9)	
Education of father				0.540§
Primary school or below	146 (9.1)	113 (9.0)	33 (9.7)	
Secondary school	1,206 (75.4)	958 (76.0)	248 (73.2)	
Tertiary school or above	247 (15.4)	189 (15.0)	58 (17.0)	
Education of mother				0.518§
Primary school or below	194 (12.2)	148 (11.7)	46 (13.6)	
Secondary school	1,190 (74.4)	938 (74.4)	252 (74.3)	
Tertiary school or above	215 (13.4)	174 (13.8)	41 (12.1)	
Other respiratory disease, yes	239 (14.9)	175 (13.9)	95 (28.0)	<0.001§
Family history of atopic disease	138 (8.6)	101 (8.0)	37 (10.9)	0.1145§

^*^Rh: rhinitis.

^†^*P*-value from Welch’s t-test between students with and without rhinitis.

^‡^*P*-value from Wilcoxon rank sum test with continuity correction between students with and without rhinitis.

^§^*P*-value from Pearson’s Chi-squared test with Yates’ continuity correction between students with rhinitis and students without rhinitis.

**Table 2:** The association between food consumption and rhinitis among primary school children.

Foods	N (Yes/No)*	Unadjusted OR_1_ (95% CI)†	Adjusted OR_2_ (95% CI)‡	Adjusted OR_3_ (95% CI)§
Meat				
<1/week	65/248	1.00	1.00	1.00
1~2/week	58/222	1.00 (0.67, 1.48)	1.30 (0.79, 2.16)	0.21 (0.03, 1.63)
≥3/week	216/790	1.04 (0.77, 1.43)	1.31 (0.84, 2.07)	1.75 (0.84, 3.84)
*P* for trend		0.755	0.321	0.214
Seafood				
<1/week	91/350	1.00	1.00	1.00
1~2/week	137/509	1.04 (0.77, 1.40)	1.21 (0.85, 1.73)	1.75 (0.86, 3.78)
≥3/week	111/401	1.06 (0.78, 1.46)	1.23 (0.85, 1.80)	1.21 (0.78, 1.92)
*P* for trend		0.695	0.324	0.506
Fruits				
<1/week	69/236	1.00	1.00	1.00
1~2/week	56/231	0.83 (0.56, 1.23)	0.94 (0.57, 1.54)	1.21 (0.76, 1.96)
≥3/week	214/793	0.92 (0.68, 1.26)	1.04 (0.68, 1.61)	0.90 (0.44, 1.91)
*P* for trend		0.767	0.67	0.749
Vegetables				
<1/week	65/229	1.00	1.00	1.00
1~2/week	39/121	1.14 (0.72, 1.78)	1.32 (0.75, 2.32)	0.97 (0.48, 2.03)
≥3/week	235/910	0.91 (0.67, 1.25)	1.03 (0.66, 1.61)	1.36 (0.62, 3.14)
*P* for trend		0.417	0.717	0.507
Beans				
<1/week	116/451	1.00	1.00	1.00
1~2/week	164/616	1.04 (0.79, 1.35)	1.10 (0.81, 1.50)	0.96 (0.45, 2.15)
≥3/week	59/193	1.19 (0.83, 1.69)	1.28 (0.86, 1.90)	1.05 (0.73, 1.52)
*P* for trend		0.384	0.228	0.974
Cereals				
<1/week	64/229	1.00	1.00	1.00
1~2/week	68/267	0.91 (0.62, 1.34)	1.09 (0.66, 1.82)	1.02 (0.64, 1.64)
≥3/week	207/764	0.97 (0.71, 1.34)	1.12 (0.70, 1.79)	1.30 (0.59, 3.05)
*P* for trend		0.954	0.674	0.989
Rice				
<1/week	76/224	1.00	1.00	1.00
1~2/week	12/66	0.54 (0.26, 1.01)	0.43 (0.20, 0.89)	1.17 (0.54, 2.68)
≥3/week	251/970	0.76 (0.57, 1.03)	0.63 (0.41, 0.97)	0.23 (0.09, 0.55)
*P* for trend		0.116	0.098	0.016
Butter				
<1/week	231/891	1.00	1.00	1.00
1~2/week	92/316	1.12 (0.85, 1.47)	1.10 (0.82, 1.46)	0.38 (0.21, 0.70)
≥3/week	16/53	1.16 (0.63, 2.03)	1.12 (0.60, 2.00)	1.03 (0.74, 1.42)
*P* for trend		0.366	0.514	0.707
Nuts				
<1/week	248/975			
1~2/week	76/256	1.17 (0.87, 1.56)	1.23 (0.90, 1.67)	0.91 (0.46, 1.72)
≥3/week	15/29	2.03 (1.05, 3.79)	2.48 (1.24, 4.76)	1.16 (0.82, 1.63)
*P* for trend		0.038	0.011	0.108
Potatoes				
<1/week	161/629	1.00	1.00	1.00
1~2/week	148/556	1.04 (0.81, 1.34)	1.07 (0.81, 1.41)	1.74 (0.81, 3.64)
≥3/week	30/75	1.56 (0.98, 2.45)	1.90 (1.16, 3.06)	1.02 (0.74, 1.40)
*P* for trend		0.156	0.048	0.378
Milk				
<1/week	95/352	1.00	1.00	1.00
1~2/week	92/417	0.82 (0.59, 1.13)	0.91 (0.62, 1.32)	1.50 (0.84, 2.62)
≥3/week	152/491	1.15 (0.86, 1.54)	1.27 (0.89, 1.81)	0.84 (0.54, 1.32)
*P* for trend		0.247	0.084	0.233
Eggs				
<1/week	81/297			
1~2/week	126523	0.88 (0.65, 1.21)	0.96 (0.65, 1.43)	1.11 (0.72, 1.73)
≥3/week	132/440	1.10 (0.81, 1.51)	1.19 (0.80, 1.78)	1.01 (0.61, 1.73)
*P* for trend		0.428	0.225	0.558
Fast food				
<1/week	207/784	1.00	1.00	1.00
1~2/week	115/419	1.04 (0.80, 1.34)	1.10 (0.83, 1.44)	1.11 (0.65, 1.92)
≥3/week	17/57	1.13 (0.63, 1.94)	1.18 (0.64, 2.08)	1.04 (0.77, 1.40)
*P* for trend		0.642	0.433	0.879

^*^N, sample size. Yes represents the number of students who had rhinitis and No represents the number of students who did not have rhinitis.

^†^OR_1_, never adjustment for any variables.

^‡^OR_2_, adjustment for age, body mass index, gender, average size of house for each member, concentration of PM_2.5_ in each school, education of father, education of mother, passive smoking at home, keeps a pet at home, has a carpet at home, has mould at home, has a plant at home, home renovation, burns incense/mosquito coils at home, other respiratory diseases, log10 (total chemical burden score), family history of atopic diseases, and weekly physical activity.

^§^OR_3_, adjustment for variable in OR_2_ as well as all foods.

**Table 3:** The association between dietary patterns and rhinitis among primary school children.

	N (Yes/No)*	Unadjusted OR_1_ (95% CI)†	Adjusted OR_2_ (95% CI)‡	Adjusted OR_3_ (95% CI)§
Pattern I				
Tertile 1	120/413	1.00	1.00	1.00
Tertile 2	109/424	0.88 (0.66, 1.19)	0.88 (0.63, 1.23)	0.93 (0.664, 1.32)
Tertile 3	110/423	0.89 (0.67, 1.2)	0.89 (0.64, 1.25)	1.02 (0.71, 1.48)
*p* for trend		0.454	0.547	0.862
Every-1 score increment	339/1,260	0.96 (0.85, 1.08)	0.97 (0.82, 1.15)	1.01 (0.85, 1.21)
Pattern II				
Tertile 1	96/437	1.00	1.00	1.00
Tertile 2	119/414	1.31 (0.97, 1.77)	1.22 (0.86, 1.71)	1.18 (0.82, 1.68)
Tertile 3	124/409	1.38 (1.02, 1.86)	1.35 (1.03, 1.84)	1.34 (1.01, 1.87)
*p* for trend		0.036	0.045	0.046
Every-1 score increment	339/1,260	1.16 (1.03, 1.3)	1.19 (1.05, 1.40)	1.19 (1.05, 1.35)
Pattern III				
Tertile 1	102/431	1.00	1.00	1.00
Tertile 2	127/406	1.32 (0.99, 1.78)	1.29 (0.92, 1.8)	1.27 (0.90, 1.80)
Tertile 3	110/423	1.1 (0.81, 1.49)	1.09 (0.80, 1.48)	1.11 (0.80, 1.54)
*p* for trend		0.549	0.599	0.561
Every-1 score increment	339/1,260	1.02 (0.9, 1.15)	1.02 (0.90, 1.15)	1.03 (0.90, 1.16)

^*^N, sample size. Yes represents the number of students who had rhinitis and No represents the number of students who did not have rhinitis.

^†^OR_1_, never adjustment for any variables.

^‡^OR_2_, adjustment for age, body mass index, gender, average size of house for each member, concentration of PM_2.5_ in each school, education of father, education of mother, passive smoking at home, keeps a pet at home, has a carpet at home, has mould at home, has a plant at home, home renovation, burns incense/mosquito coils at home, other respiratory diseases, log10 (total chemical burden score), family history of atopic diseases, and weekly physical activity.

^§^OR_3_, adjustment for variable in OR_2_ as well as three patterns.
