# Supplementary material for: Dietary patterns and the risk of rhinitis in primary school children: a prospective cohort study
Source: Sci Rep. 2017 Mar 15;7:44610. doi: 10.1038/srep44610 (PMC5353568; doi:10.1038/srep44610)
Supplement: Supplementary Information [file srep44610-s1.doc]

Dietary patterns and the risk of rhinitis in primary school children: a prospective cohort study

Xudong Liu^1^, Claudie Chiu-Yi Wong^1^, Ignatius T.S. Yu^1,3^, Zilong Zhang^1^, Lixing Tan^1^, Arthur P.S. Lau^2^, Albert Lee^1^, Eng Kiong YEOH ^1^, Xiang Qian Lao ^1^,*

1. JC School of Public Health and Primary Care, The Chinese University of Hong Kong, Hong Kong SAR, China;

2. Division of Environment, Hong Kong University of Science and Technology, Hong Kong SAR, China;

3. Hong Kong Occupational and Environmental Health Academy, Hong Kong SAR, China

* Address correspondence to: Xiang Qian Lao, Ph.D., Assistant Professor; Institute: JC School of Public Health and Primary Care, The Chinese University of Hong Kong; Address: 4/F School of Public Health, Prince of Wales Hospital, Sha Tin, N.T., Hong Kong SAR, China. Telephone number: +852 22528763. Fax: +852 26063500. E-mail:  [xqlao@cuhk.edu.hk](mailto:xqlao@cuhk.edu.hk)

Table S1. Rotated factor loading based on thirteen groups of food ^*^

|  | Pattern I | Pattern II | Pattern III | Communality | Uniqueness | KMO |
| --- | --- | --- | --- | --- | --- | --- |
| Meat | 0.83 | 0.12 | 0.12 | 0.71 | 0.29 | 0.95 |
| Seafood | 0.66 | 0.22 | 0.04 | 0.49 | 0.51 | 0.96 |
| Fruits | 0.84 | 0.20 | 0.02 | 0.74 | 0.26 | 0.94 |
| Vegetables | 0.89 | 0.13 | 0.04 | 0.80 | 0.20 | 0.92 |
| Beans | 0.51 | 0.54 | 0.01 | 0.55 | 0.45 | 0.95 |
| Cereals | 0.81 | 0.18 | 0.17 | 0.72 | 0.28 | 0.95 |
| Rice | 0.86 | 0.08 | 0.12 | 0.77 | 0.23 | 0.94 |
| Butter | 0.11 | 0.55 | 0.43 | 0.51 | 0.50 | 0.89 |
| Nuts | 0.09 | 0.84 | 0.03 | 0.71 | 0.29 | 0.86 |
| Potatoes | 0.28 | 0.66 | 0.19 | 0.55 | 0.45 | 0.91 |
| Milk | 0.61 | 0.26 | 0.21 | 0.51 | 0.52 | 0.95 |
| Eggs | 0.66 | 0.27 | 0.20 | 0.55 | 0.45 | 0.95 |
| Fast food | 0.15 | 0.11 | 0.91 | 0.87 | 0.13 | 0.91 |
| SS loadings | 5.20 | 2.04 | 1.20 |  |  |  |
| Proportion Variation | 0.40 | 0.16 | 0.09 |  |  |  |
| Cumulative Variation | 0.40 | 0.56 | 0.65 |  |  |  |
| Proportion Explained | 0.62 | 0.24 | 0.14 |  |  |  |
| Cumulative Proportion | 0.62 | 0.86 | 1.00 |  |  |  |

^*^ The p-value for Bartlett's test of sphericity was < 0·001, the overall value of Kaiser–Meyer–Olkin (KMO) statistic was 0.94, and the individual measures of sampling adequacy for each item ranged from 0.86 (nuts) to 0.96 (seafood).

Table S2. Sensitivity analysis on the association between dietary patterns and rhinitis by removing subjects with other respiratory diseases

|  | N (Yes/No) ^*^ | OR_1_ (95% CI)^†^ | OR_2_ (95% CI) ^‡^ | OR_3_ (95% CI) ^§^ |
| --- | --- | --- | --- | --- |
| Pattern I |  |  |  |  |
| Tertile 1 | 84/369 | 1.00 | 1.00 | 1.00 |
| Tertile 2 | 84/354 | 1.04 (0.74, 1.46) | 1.06 (0.73, 1.54) | 1.14 (0.77, 1.68) |
| Tertile 3 | 76/362 | 0.92 (0.65, 1.3) | 0.94 (0.64, 1.38) | 1.11 (0.73, 1.69) |
| *p* for trend |  | 0.650 | 0.697 | 0.789 |
| Every-1 score increment | 244/1,085 | 1 (0.87, 1.15) | 1.02 (0.84, 1.25) | 1.08 (0.88, 1.33) |
| Pattern II |  |  |  |  |
| Tertile 1 | 67/384 | 1.00 | 1.00 | 1.00 |
| Tertile 2 | 82/357 | 1.32 (0.93, 1.88) | 1.31 (0.88, 1.95) | 1.30 (0.85, 1.96) |
| Tertile 3 | 95/344 | 1.58 (1.12, 2.24) | 1.59 (1.12, 2.27) | 1.59 (1.11, 2.28) |
| *p* for trend |  | 0.009 | 0.010 | 0.009 |
| Every-1 score increment | 244/1,085 | 1.21 (1.06, 1.38) | 1.24 (1.08, 1.42) | 1.25 (1.09, 1.43) |
| Pattern III |  |  |  |  |
| Tertile 1 | 74/369 | 1.00 | 1.00 | 1.00 |
| Tertile 2 | 88/353 | 1.24 (0.88, 1.75) | 1.26 (0.86, 1.84) | 1.25 (0.84, 1.87) |
| Tertile 3 | 82/363 | 1.13 (0.8, 1.59) | 1.1 (0.78, 1.57) | 1.13 (0.78, 1.63) |
| *p* for trend |  | 0.508 | 0.583 | 0.513 |
| Every-1 score increment | 244/1,085 | 1.03 (0.9, 1.18) | 1.03 (0.89, 1.19) | 1.04 (0.90, 1.20) |

^*^ N, sample size. Yes represents the number of students who had rhinitis and No represents the number of students who did not have rhinitis.

^†^ OR_1_, never adjustment for any variables

^‡^ OR_2_, adjustment for age, body mass index, gender, average size of house for each member, concentration of PM_2.5_ in each school, education of father, education of mother, passive smoking at home, keeps a pet at home, has a carpet at home, has mould at home, has a plant at home, home renovation, burns incense/mosquito coils at home, log10 (total chemical burden score), family history of atopic diseases, and weekly physical activity.

^§^ OR_3_, adjustment for variable in OR_2_ as well as three patterns

Table S3. Sensitivity analysis on the association between dietary patterns and rhinitis by removing subjects with family history of atopic diseases

|  | N (Yes/No) ^*^ | OR_1_ (95% CI)^†^ | OR_2_ (95% CI) ^‡^ | OR_3_ (95% CI) ^§^ |
| --- | --- | --- | --- | --- |
| Pattern I |  |  |  |  |
| Tertile 1 | 113/396 | 1.00 | 1.00 | 1.00 |
| Tertile 2 | 95/379 | 0.88 (0.65, 1.19) | 0.89 (0.63, 1.27) | 0.95 (0.66, 1.35) |
| Tertile 3 | 94/384 | 0.86 (0.63, 1.17) | 0.86 (0.6, 1.23) | 1.00 (0.68, 1.46) |
| *p* for trend |  | 0.322 | 0.423 | 0.775 |
| Every-1 score increment | 302/1,159 | 0.94 (0.83, 1.06) | 0.97 (0.81, 1.16) | 1.01 (0.84, 1.22) |
| Pattern II |  |  |  |  |
| Tertile 1 | 80/391 | 1.00 | 1.00 | 1.00 |
| Tertile 2 | 112/400 | 1.37 (1.00, 1.89) | 1.21 (0.84, 1.74) | 1.17 (0.79, 1.70) |
| Tertile 3 | 110/368 | 1.46 (1.06, 2.02) | 1.43 (1.03, 2.00) | 1.43 (1.02, 2.02) |
| *p* for trend |  | 0.022 | 0.033 | 0.036 |
| Every-1 score increment | 302/1,159 | 1.17 (1.03, 1.32) | 1.2 (1.05, 1.37) | 1.20 (1.05, 1.37) |
| Pattern III |  |  |  |  |
| Tertile 1 | 87/393 | 1.00 | 1.00 | 1.00 |
| Tertile 2 | 119/383 | 1.4 (1.03, 1.92) | 1.31 (0.92, 1.86) | 1.29 (0.89, 1.86) |
| Tertile 3 | 96/383 | 1.13 (0.82, 1.56) | 1.12 (0.81, 1.56) | 1.13 (0.80, 1.60) |
| *p* for trend |  | 0.463 | 0.495 | 0.492 |
| Every-1 score increment | 302/1,159 | 1.03 (0.9, 1.16) | 1.04 (0.91, 1.19) | 1.04 (0.91, 1.19) |

^*^ N, sample size. Yes represents the number of students who had rhinitis and No represents the number of students who did not have rhinitis.

^†^ OR_1_, never adjustment for any variables

^‡^ OR_2_, adjustment for age, body mass index, gender, average size of house for each member, concentration of PM_2.5_ in each school, education of father, education of mother, passive smoking at home, keeps a pet at home, has a carpet at home, has mould at home, has a plant at home, home renovation, burns incense/mosquito coils at home, other respiratory diseases, log10 (total chemical burden score), and weekly physical activity.

^§^ OR_3_, adjustment for variable in OR_2_ as well as three patterns

Table S4. The association between dietary patterns and rhinitis based on median of age

|  | N (Yes/No) ^*^ | Unadjusted  OR_1_ (95% CI)^†^ | Adjusted  OR_2_ (95% CI) ^‡^ | Adjusted  OR_3_ (95% CI) ^§^ |
| --- | --- | --- | --- | --- |
| Age ≤ 9 years ^#^ |  |  |  |  |
| Pattern I |  |  |  |  |
| Tertile 1 | 42/156 | 1.00 | 1.00 | 1.00 |
| Tertile 2 | 79/295 | 0.99 (0.66, 1.53) | 1.02 (0.66, 1.58) | 1.01 (0.65, 1.58) |
| Tertile 3 | 83/311 | 0.99 (0.66, 1.52) | 0.94 (0.61, 1.47) | 1.01 (0.64, 1.61) |
| *p* for trend |  | 0.967 | 0.744 | 0.950 |
| Every-1 score increment | 204/762 | 1.13 (0.01, 139.98) | 1.13 (0.86, 1.51) | 1.33 (0.97, 1.86) |
| Pattern II |  |  |  |  |
| Tertile 1 | 73/307 | 1.00 | 1.00 | 1.00 |
| Tertile 2 | 43/176 | 1.03 (0.67, 1.56) | 1.03 (0.67, 1.59) | 1.05 (0.67, 1.61) |
| Tertile 3 | 88/279 | 1.33 (0.94, 1.89) | 1.3 (0.91, 1.87) | 1.31 (0.90, 1.90) |
| *p* for trend |  | 0.112 | 0.151 | 0.150 |
| Every-1 score increment | 204/762 | 3.54 (0.21, 63.35) | 1.21 (1.04, 1.4) | 1.26 (1.08, 1.47) |
| Pattern III |  |  |  |  |
| Tertile 1 | 81/311 | 1.00 | 1.00 | 1.00 |
| Tertile 2 | 47/180 | 1 (0.67, 1.5) | 0.98 (0.64, 1.47) | 0.98 (0.64, 1.48) |
| Tertile 3 | 76/271 | 1.08 (0.76, 1.53) | 1.08 (0.75, 1.54) | 1.08 (0.74, 1.59) |
| *p* for trend |  | 0.684 | 0.693 | 0.650 |
| Every-1 score increment | 204/762 | 1.65 (0.11, 24.46) | 1.04 (0.89, 1.2) | 1.10 (0.94, 1.29) |
| Age > 9 years |  |  |  |  |
| Pattern I |  |  |  |  |
| Tertile 1 | 78/257 | 1.00 | 1.00 | 1.00 |
| Tertile 2 | 30/129 | 0.77 (0.47, 1.22) | 0.90 (0.49, 1.64) | 1.04 (0.55, 1.94) |
| Tertile 3 | 27/112 | 0.79 (0.48, 1.28) | 1.04 (0.55, 1.92) | 1.34 (0.67, 2.67) |
| *p* for trend |  | 0.27 | 0.163 | 0.644 |
| Every-1 score increment | 135/498 | 1.54 (0.11, 21.95) | 1.04 (0.79, 1.39) | 1.08 (0.81, 1.44) |
| Pattern II |  |  |  |  |
| Tertile 1 | 23/130 | 1.00 | 1.00 | 1.00 |
| Tertile 2 | 76/238 | 1.80 (1.10, 3.07) | 1.35 (0.69, 2.67) | 1.30 (0.61, 2.73) |
| Tertile 3 | 36/130 | 1.57 (0.88, 2.82) | 1.44 (0.77, 2.73) | 1.45 (0.75, 2.87) |
| *p* for trend |  | 0.935 | 0.257 | 0.819 |
| Every-1 score increment | 135/498 | 0.74 (0.21, 59.46) | 1.18 (0.93, 1.51) | 1.12 (1.01, 1.53) |
| Pattern III |  |  |  |  |
| Tertile 1 | 21/120 | 1.00 | 1.00 | 1.00 |
| Tertile 2 | 80/226 | 2.02 (1.21, 3.5) | 1.73 (0.88, 3.47) | 1.76 (0.86, 3.64) |
| Tertile 3 | 34/152 | 1.28 (0.71, 2.34) | 1.11 (0.58, 2.13) | 1.21 (0.62, 2.37) |
| *p* for trend |  | 0.633 | 0.218 | 0.720 |
| Every-1 score increment | 135/498 | 0.42 (0.01, 29.21) | 1.03 (0.8, 1.32) | 1.04 (0.80, 1.33) |

^*^ N, sample size. Yes represents the number of students who had rhinitis and No represents the number of students who did not have rhinitis.

^†^ OR_1_, never adjustment for any variables

^‡^ OR_2_, adjustment for age, body mass index, gender, average size of house for each member, concentration of PM_2.5_ in each school, education of father, education of mother, passive smoking at home, keeps a pet at home, has a carpet at home, has mould at home, has a plant at home, home renovation, burns incense/mosquito coils at home, other respiratory diseases, log10 (total chemical burden score), family history of atopic diseases, and weekly physical activity.

^§^ OR_3_, adjustment for variable in OR_2_ as well as three patterns.

^#^ The groups were categorized based on the median of age of all students (9 years).

Table S5. The association between dietary pattern and rhinitis based on gender

|  | N  (Yes/No) ^*^ | Unadjusted  OR_1_ (95% CI)^†^ | Adjusted  OR_2_ (95% CI) ^‡^ | Adjusted  OR_3_ (95% CI) ^§^ |
| --- | --- | --- | --- | --- |
| Males |  |  |  |  |
| Pattern I |  |  |  |  |
| Tertile 1 | 51/215 | 1.00 | 1.00 | 1.00 |
| Tertile 2 | 51/171 | 1.26 (0.81, 1.95) | 1.09 (0.66, 1.80) | 1.10 (0.66, 1.86) |
| Tertile 3 | 58/192 | 1.27 (0.83, 1.95) | 1.08 (0.66, 1.77) | 1.28 (0.75, 2.20) |
| *p* for trend |  | 0.264 | 0.773 | 0.353 |
| Every-1 score increment |  | 1.15 (0.97, 1.38) | 1.11 (0.85, 1.44) | 1.18 (0.90, 1.55) |
| Pattern II |  |  |  |  |
| Tertile 1 | 49/197 | 1.00 | 1.00 | 1.00 |
| Tertile 2 | 48/204 | 0.95 (0.61, 1.48) | 1.00 (0.59, 1.68) | 1.03 (0.59, 1.76) |
| Tertile 3 | 63/177 | 1.43 (0.94, 2.20) | 1.50 (0.96, 2.36) | 1.58 (0.99, 2.54) |
| *p* for trend |  | 0.093 | 0.077 | 0.051 |
| Every-1 score increment |  | 1.23 (1.04, 1.45) | 1.24 (1.03, 1.48) | 1.26 (1.05, 1.51) |
| Pattern III |  |  |  |  |
| Tertile 1 | 49/196 | 1.00 | 1.00 | 1.00 |
| Tertile 2 | 57/201 | 1.13 (0.74, 1.75) | 1.19 (0.73, 1.93) | 1.28 (0.77, 2.14) |
| Tertile 3 | 54/181 | 1.19 (0.77, 1.85) | 1.15 (0.73, 1.81) | 1.23 (0.76, 2.00) |
| *p* for trend |  | 0.427 | 0.548 | 0.376 |
| Every-1 score increment |  | 1.06 (0.89, 1.26) | 1.03 (0.86, 1.23) | 1.05 (0.87, 1.26) |
| Females |  |  |  |  |
| Pattern I |  |  |  |  |
| Tertile 1 | 69/198 | 1.00 | 1.00 | 1.00 |
| Tertile 2 | 58/253 | 0.66 (0.44, 0.98) | 0.72 (0.46, 1.14) | 0.77 (0.48, 1.23) |
| Tertile 3 | 52/231 | 0.65 (0.43, 0.97) | 0.71 (0.44, 1.15) | 0.79 (0.47, 1.34) |
| *p* for trend |  | 0.033 | 0.19 | 0.288 |
| Every-1 score increment |  | 0.81 (0.69, 0.95) | 0.87 (0.69, 1.1) | 0.89 (0.704, 1.14) |
| Pattern II |  |  |  |  |
| Tertile 1 | 47/240 | 1.00 | 1.00 | 1.00 |
| Tertile 2 | 71/210 | 1.73 (1.15, 2.62) | 1.47 (0.92, 2.37) | 1.36 (0.83, 2.23) |
| Tertile 3 | 61/232 | 1.34 (0.88, 2.05) | 1.33 (0.86, 2.08) | 1.28 (0.82, 2.03) |
| *p* for trend |  | 0.192 | 0.207 | 0.303 |
| Every-1 score increment |  | 1.10 (0.93, 1.29) | 1.17 (0.98, 1.39) | 1.15 (0.96, 1.37) |
| Pattern III |  |  |  |  |
| Tertile 1 | 53/235 | 1.00 | 1.00 | 1.00 |
| Tertile 2 | 70/205 | 1.51 (1.01, 2.27) | 1.29 (0.80, 2.06) | 1.17 (0.71, 1.91) |
| Tertile 3 | 56/242 | 1.03 (0.68, 1.56) | 1.05 (0.68, 1.62) | 1.02 (0.65, 1.61) |
| *p* for trend |  | 0.925 | 0.836 | 0.975 |
| Every-1 score increment |  | 0.98 (0.83, 1.16) | 1.02 (0.85, 1.22) | 1.02 (0.85, 1.21) |

^*^ N, sample size. Yes represents the number of students who had rhinitis and No represents the number of students who did not have rhinitis.

^†^ OR_1_, never adjustment for any variables

^‡^ OR_2_, adjustment for age, body mass index, average size of house for each member, concentration of PM_2.5_ in each school, education of father, education of mother, passive smoking at home, keeps a pet at home, has a carpet at home, has mould at home, has a plant at home, home renovation, burns incense/mosquito coils at home, other respiratory diseases, log10 (total chem*i*cal burden score), family history of atopic diseases, and weekly physical activity.

^§^ OR_3_, adjustment for variable in OR_2_ as well as three patterns.
